# Effect of sildenafil citrate on endometrial preparation and outcome of frozen-thawed embryo transfer cycles: a randomized clinical trial

**Published:** 2013-02

**Authors:** Razieh Dehghani Firouzabadi, Robab Davar, Farzaneh Hojjat, Mohamad Mahdavi

**Affiliations:** 1 *Department of Obstetrics and Gynecology, Research and Clinical Center for Infertility, Shahid Sadoughi University of Medical Sciences, Yazd, Iran.*; 2 *Shahid Beheshti Taft Hospital, Shahid Sadoughi University of Medical Sciences, Yazd, Iran.*

**Keywords:** *Sildenafil citrate*, *Endometrial thickness*, *Embryo transfer*

## Abstract

**Background:** Sildenafil citrate may increase endometrial thickness and affect the outcome of frozen-thawed embryo transfer cycles.

**Objective:** The aim of this study was to estimate the effect of sildenafil citrate on ultrasonographic endometrial thickness and pattern and to investigate the estrogen level on the day of progesterone administration, the implantation rate and chemical pregnancy rate in frozen embryo transfer cycles.

**Materials and Methods:** This randomized controlled trial was conducted on 80 patients who had an antecedent of poor endometrial response and frozen embryos. 40 patients were given estradiol by a step up method with menstruation to prepare the endometrium, and the other 40 were given sildenafil citrate tablets (50 mg) daily in addition to the above treatment protocol from the first day of the cycle until the day progesterone was started. This was discontinued 48-72 hours prior to the embryo transfer.

**Results:** The endometrial thickness was significantly higher in the sildenafil citrate group (p<0.0001), the triple line patterns of the endometrium were significantly higher in the sildenafil citrate group (p<0.0001), while the intermediate patterns of the endometrium were not significantly different in the two groups. The echogen patterns of the endometrium were significantly higher in control group (p<0.0001). Finally, implantation rate and the chemical pregnancy rates were higher in the sildenafil citrate group but not significantly.

**Conclusion:** As our study shows, the oral use of sildenafil citrate is a good way to improve the endometrial receptivity. We recommend the routine use of oral sildenafil citrate in patients with a previous failure of assisted reproduction technology cycles due to poor endometrial thickness.

## Introduction

Despite the improvement in ovarian stimulation protocols and subsequent access to a number of dominant follicles, mature oocytes, and embryos for transfer, in vitro fertilization (IVF) has successfully reached a plateau. Therefore, more attention should be focused on implantation and endometrial receptivity. Implantation remains a major limiting step of assisted reproductive technology (ART) and uterine receptivity is essential for successful implantation in all species (1). 

Successful implantation requires good embryo quality, appropriately timed and arranged endometrial receptivity, and efficient crosstalk between the embryo and the receptive endometrium. It is thought that the impairment of any one of these factors or biological processes may result in implantation failure (2). 

The endometrium is normally a non-receptive environment for an embryo, except during the window. Implantation window is a period during which the endometrium is optimally receptive to implanting blastocyst within the cycle days 20 and 24. It is characterized by a refractory endometrial status (3). Endometrial receptivity during the implantation window depends on the following factors (4-13).

**Morphological Markers**

1. Endometrial thickness
2. Endometrial echogenic pattern
3. Endometrial and sub endometrial blood flows

During implantation window, the endometrial epithelium encompasses four cell types: microvilli-rich cells, pinopode cells, vesiculated cells, and ciliated cells (14). 

**Biochemical Markers**

1. Endometrial adhesion molecules
2. Endometrial anti-adhesion molecules
3. Endometrial cytokines
4. Growth factors
5. Endometrial immune markers
6. Endometrial Glycodelin
7. Insulin like growth factor (IGF)
8. Leukemia inhibitory factor (LIF).

**The process of embryo implantation is described as having three phases**

1. Apposition: “unstable adhesion” of the transferred embryo to the surface of the uterine lining.
2. Attachment (adhesion): “stable adhesion,” believed to involve signaling back and forth between the embryo and the lining.
3. Penetration (invasion): invasion of the trophectoderm cells from the embryo through the surface of the lining deeper the stroma of the uterine lining, forming a vascular connection to the mother (15).

In any of these stages, a number of the above factors are effective. There are 14 treatment options for improving the implantation:

(a) blastocyst transfer, (b) assisted hatching, (c) co-culture, (d) preimplantation genetic screening, (e) hysteroscopy, (f) sildenafil citrate, (g) salpingectomy for tubal disease, (h) oocyte donation, (i) transfer of six or more embryos, (j) intratubal embryo transfer, (k) natural-cycle IVF, (l) antiphospholipid antibodies (APA) testing and treatment, (m) allogenic lymphocyte therapy, and (n) IV immunoglobin therapy (15). 

In this article, we have focused on endometrial thinning as a cause of implantation failure, and discuss its causes and treatment methods.

**Causes of thin endometrium**

1. Permanent damage to the basal endometrium.
2. Endometrial resistance to estrogen.
3. Reduced blood flow.
4. Over-exposure to testosterone.

Estrogen induced endometrial proliferation is in large part dependent upon blood flow to the basal endometrium (16).

Nitric oxide (NO) leads to relaxation of vascular smooth muscles through a cyclic guanyl monophosphate (cGMP) mediated pathway. Nitric oxide synthase isoforms have been identified in the vascular muscles of both human endometrium and myometrium. Phosphodiesterase (PDE) is a family of isoenzymes that hydrolyze cyclic nucleotides, such as cGMP. The inhibitors of specific Phosphodiesterase (PDE) subtypes have been identified with an ability to augment the effects of cyclic nucleotides on target tissues as the endometrium    (16) .

Sildenafil citrate is a potent and selective inhibitor of cGMP specific phosphodiesterase type 5 (PDE5) that prevents the breakdown of cGMP and potentiates the effect of nitric oxide on vascular smooth muscles (17). Sildenafil citrate could lead to an improvement in uterine blood flow and, in conjunction with estrogen, led to the estrogen-induced proliferation of the endometrial lining (16).

Sildenafil citrate enhances uterine blood flow and increases endometrial thickening (18). The achieved implantation depends on the blastocyst’s ability to infiltrate the endometrium and develop a sustaining blood supply, which requires the following genes to produce the necessary proteins for digesting the endometrial cellular matrix, to regulate cell growth, and to induce angiogenesis:

a) Tumor suppressor factor (p53).

b) Plasminogen activator inhibitor 1 (PAI-1).

c) Vascularendothelial growth factor (VEGF) (15, 18, 19).

Sildenafil citrate was enhanced markedly in p53 and stimulated angiogenic responses with increased VEGF (20, 21). The aim of this study was to estimate the effect of sildenafil citrate on ultrasonographic endometrial thickness and pattern and to investigate the estrogen level on the day of progesterone administration, the implantation rate and chemical pregnancy rate in frozen embryo transfer cycles.

## Materials and methods

**Subjects**

The study was a randomized clinical controlled trial, conducted in the Research and Clinical Center for Infertility affiliated to Shahid Sadoughi University of Medical Sciences between 2009-2011. The project was approved by the ethical committee of the infertility department of the university and was initiated after getting written consents from the participants and the financial support from the university. A total of 80 patients with an antecedent of poor endometrial response and frozen embryos were included in this study.

The inclusion criteria required the subjects to :

1. be younger than 40 years.
2. have high-quality frozen embryos.

The exclusion criteria included:

1. a history of endocrine diseases.
2. a history of hysteroscopic surgeries.
3. cardiovascular, renal and liver diseases.
4. hypotension (blood pressure <90/50 mmHg).
5. a history of stroke or myocardial infarction.

**Treatment protocols**

Patients who met these conditions entered the study and were divided into two groups based on randomized tables. To prepare the endometrium, one of the two groups was given estradiol by a step-up method while in menstruation. From the first to the fourth day of the menstrual cycle, 2 mg estradiol valerat tablets, from the 5^th^ to the 8^th^ day of the menstrual cycle, 4 mg estradiol valerat tablets, and from the 9^th^ to the 12^th^ day of the menstrual, 6 mg estradiol valerat tablets were given daily. The second group was given sildenafil citrate tablets (50 mg) daily in addition to the above treatment protocol from the first day of the cycle until the day of starting progesterone. It was discontinued 48-72 hours prior to the embryo transfer. 

**Assays**

On the thirteenth day of the menstrual cycle, the endometrial thickness was estimated by transvaginal ultrasonography (arranged in a series every other day). The evaluations were performed by a single investigator. If the endometrial thickness was more than 8mm, 100 mg of progesterone was injected intramuscularly. Then estrogen and progesterone were measured in the blood samples in an hour, and, after three days, embryos were transferred.

**Outcome**

Administering stradiol valerat and progesterone continued until two weeks after the embryos were transferred. In case BHCG was tested and proved to be positive, estradiol valerat and progesterone were continueed until the 11^th^ week of pregnancy. Then, four weeks after the embryo transfer, the number of gestational sacs were determined by vaginal ultrasound. 

**Statistical analysis**

After data collection and coding, the data were entered into the computer. Using version 18 of SPSS software, Mann-Whitney test, Chi-square, and independent-samples T-test, the results were analyzed. 

## Results

The patients were divided into two groups based on randomized tables. The basal patients’ variables are shown in Table I. There was no significant difference between the two groups. Long cycle, estrogen, progesterone, and estrogen/ progesterone ratio on the progesterone day was significantly different in the two groups, but LH on the progesterone day was not significantly different in the two groups (Table II). Endometrial thickness was significantly higher in the sildenafil citrate group (p<0.0001). 

Implantation rate was higher in the sildenafil citrate group but not significantly (p=0.22). The triple line pattern of the endometrium was significantly higher in the sildenafil citrate group (p<0.0001), while the intermediate pattern of the endometrium was not significantly different in the two groups, the echogen pattern of the endometrium was significantly higher in the control group (p<0.0001) (Table III). Finally chemical pregnancy rates were higher in the sildenafil citrate group but not significantly (Figure I, II).

**Table I:** Characteristics of patients at the beginning of the cycle

**Variable**	**Control group (mean)**	**Sildenafil citrate group (mean)**	**p-value**
Infertility duration (months)	71	75	0.39
Age (years)	28	29	0.47
Basal FSH ( IU/L)	5.4	5.6	0.4
Basal LH ( IU/L)	8.765	8.77	1
Basal estrogen (pg/mL)	55.8	56.5	0.73
Basal progesterone (ng/ml)	0.66	0.645	0.7
Basal FSH/LH	0.7	0.72	0.7

**Table II:** Effect of sildenafil citrate on long cycle, progesterone, estrogen, estrogen/ progesterone and LH on progesterone day, endometrial thickness, and implantation rate

**Variable**	**Control group (mean)**	**Sildenafil citrate group (mean)**	**p-value**
Duration cycle ( days)	13.375	12.125	<0.0001
Progesterone on progesterone day (ng/mL)	0.88	1.12	<0.0001
Estrogen on progesterone day (pg/mL)	984.5	643.5	<0.0001
Estrogen/ progesterone on progesterone day	0.9	0.73	<0.0001
LH on progesterone day (IU/L)	1.13	1.1	0.98
Endometrial thickness (mm)	8.0	9.8	<0.0001
Implantation rate (%)	8.75	14.16	0.22

**Table III:** Effect of FSH/LH ratio on endometrial pattern and chemical pregnancy

**Variable** ** (mm)**	**Control group**		**Sildenafil citrate group**		**p-value**
	**+**	**-**	**+**	**-**	
Triple line	12 (30%)	28 (70%)	31 (77.5%)	9 (22.5%)	<0.0001
Intermediate	1 (2.5%)	39 (97.5%)	3 (7.5%)	37 (92.5%)	0.3
Echogen	26 (65%)	14 (35%)	6 (15%)	34 (85%)	<0.0001
Chemical pregnancy	8 (20%)	32 (80%)	13 (32%)	27 (68%)	0.13

**Figure 1:**
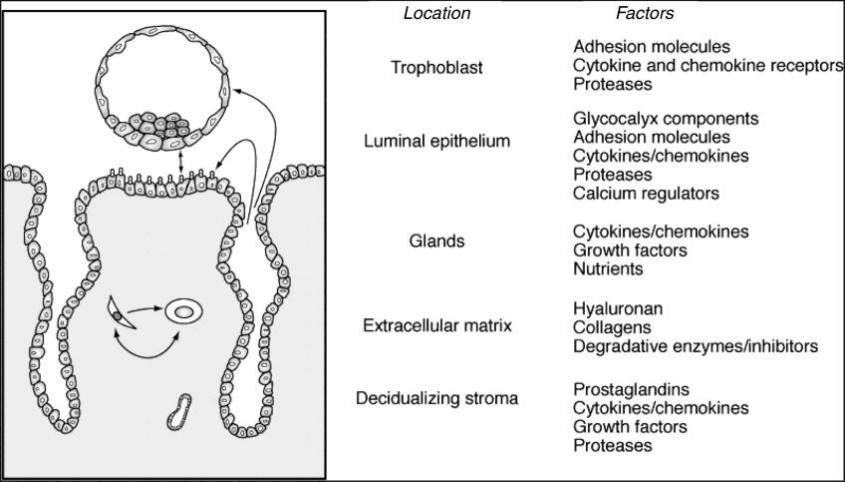
Factors regulated during the early stages of implantation. (Diedrich *et al* Hum Reprod Update 2007; 13: 365-377)

**Figure 2:**
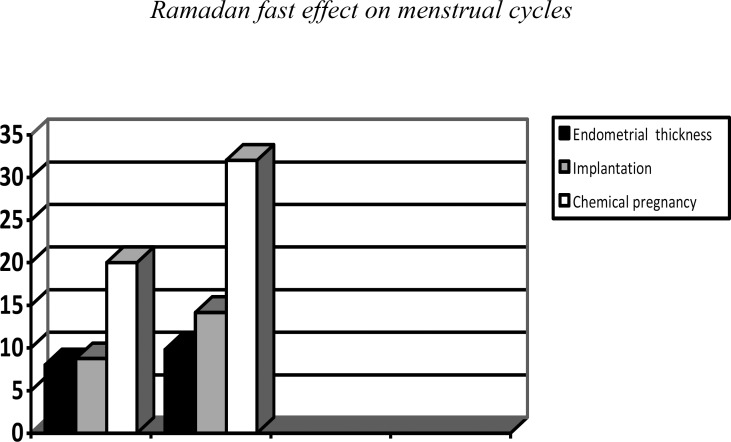
Comparison of endometrial thickness, implantation, and chemical pregnancy in the two groups

**Figure 3:**
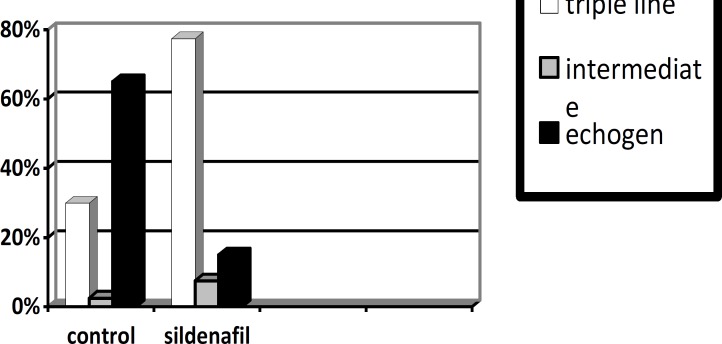
Comparison of echogen, intermediate and triple line pattern of endometrium in the two groups

**Figure 4:**
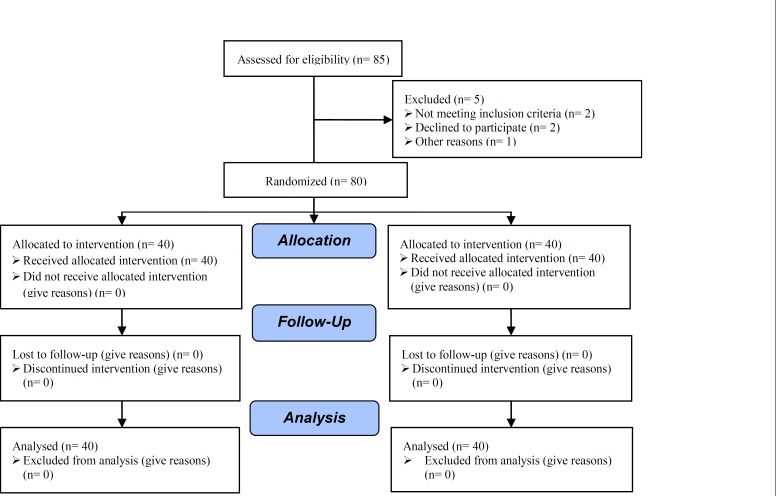
Consort flow diagram

## Discussion

In this study, we evaluated the effect of sildenafil citrate on the outcome of frozen-thawed embryo transfer cycles. Endometrial growth seems to be dependent on uterine artery blood flow, and the importance of endometrial development in the pregnancy outcome has been already reported (22, 23). A positive linear correlation between endometrial thickness measured on the hCG day and pregnancy rate (PR) was identified (24). A good correlation has been found between endometrial thickness and the prevalence of conception. An endometrial thickness of ≥9 mm in the late proliferative phase, as determined by vaginal ultrasound, correlates well with the chance of pregnancy after IVF, whereas a thinner endometrium is associated with poorer prognosis for success (23). 

Sildenafil citrate improves the uterine artery blood flow and the sonographic endometrial thickening in patients with a prior assisted reproductive cycle failing due to poor endometrial response (16, 23, 25). Increased endometrial thickness is associated with higher pregnancy rates. However, no attainment of pregnancy was predicted by endometrial thickness alone (26). In our study, sildenafil citrate clearly increased endometrial thickness (9.8 mm vs. 8 mm), and the chemical pregnancy rates were higher in the sildenafil citrate group but not significantly.

Uterine receptivity was assessed simultaneously by measuring vasoactive cytokines possibly involved in the development of spiral arteries and assessing endometrial and uterine arterial blood flow (1). Endometrial and subendometrial blood flows measured by vaginal color Doppler ultrasound are a good predicator of pregnancy during IVF treatment (6, 27). The present results confirm and extend previous observations that the advanced hyperechogenic transformation of the endometrium is associated with poor in vitro fertilization and embryo transfer (IVF-ET) outcome (28).

In our study, sildenafil citrate increased the triple line pattern of the endometrium but decreased its echogen pattern. A combined analysis of endometrial thickness and pattern on the day of hCG administration proved to be a better predictor of the outcome of IVF/ICSI-ET and may be more helpful for patient counseling than a separate analysis (29). Elevated estrogen concentrations may increase sensitivity to progesterone action and, thus, lead to secretory advancement (30). Estrogen/progesterone ratios, which are also associated with the impairment of endometrial receptivity, are the main factors affecting receptivity (31). In this study, sildenafil citrate significantly decreased the estrogen, progesterone and estrogen/ progesterone ratio on the progesterone day.

## Conclusion

In our study, it was shown that the oral use of sildenafil citrate can be a good way to improve endometrial receptivity. We recommend the routine use of oral sildenafil citrate in patients with a previous failure of assisted reproduction technology cycles due to poor endometrial thickness.
